# Glenoid morphology variation between patients with hypermobile shoulder joints and controls: Identification of hyperlaxity-related morphologic bone changes

**DOI:** 10.1016/j.redii.2024.100052

**Published:** 2024-08-23

**Authors:** Sirine Hamitouche, Fatma Boubaker, Gabriela Hossu, François Sirveaux, Romain Gillet, Alain Blum, Pedro Augusto Gondim Teixeira

**Affiliations:** a Guilloz Imaging Department, Hôpital Central, Centre Hospitalier Universitaire de Nancy, 29, avenue du Maréchal-de-Lattre-de-Tassigny, 54035 Nancy Cedex, France; b Université de Lorraine, Inserm, Iadi, 54000 Nancy, France; c Department of Hand Surgery, Plastic and Reconstructive Surgery, Centre Chirurgical Émile-Gallé, Centre Hospitalier Universitaire de Nancy, Nancy, France

**Keywords:** Shoulder joint, Joint hypermobility, Computed tomography, Glenoid cavity, Diagnostic imaging

## Abstract

**Objective:**

Our study aims to quantitatively determine the concavity of the glenoid articular surface in patients with hypermobile shoulders compared to those without.

**Method:**

We examined medical records of shoulder CTs from 2017 to 2022, selecting 50 patients with clinical signs of joint hypermobility for our case group and 54 for our control group. Two blinded readers independently assessed the glenoid morphology, calculating the glenoid concavity angle (GCA) and evaluating the articular surface shape as concave, flat, or convex. They also recorded the presence and severity of glenoid dysplasia. We compared these assessments between groups.

**Results:**

The mean GCA was significantly lower in the hypermobile group (2.3 ± 3.7° and 2.3 ± 3.8°) versus controls (6.6 ± 3.3° and 5.3 ± 3.8°) (*P* < 0.05). Interobserver reproducibility was high (ICC=0.76). A stark difference in glenoid morphology was noted between groups (*P* < 0.001), with a majority of hypermobile patients having a flat or convex glenoid. GCAs decreased with increasing shoulder laxity and dysplasia. GCA showed 77–81 % sensitivity and 55–82 % specificity for detecting shoulder hyperlaxity with a 4° cutoff.

**Conclusion:**

There is a significant association between GCA and shoulder hyperlaxity, demonstrating diagnostic efficacy and substantial interobserver agreement.

**Clinical Relevance:**

GCA values lower than 4° warrant further clinical investigation for shoulder hyperlaxity and associated conditions, which is crucial for patient treatment planning.

## Key points

• Shoulder hyperlaxity is underdiagnosed with potential implications in patient care.
• The glenoid concavity is significantly smaller in hypermobile shoulders.
• The glenoid concavity angle has a good diagnostic performance for hyperlaxity.
• Low glenoid concavity angles (< 4°) should warrant a search for shoulder hyperlaxity.

## 1 Introduction

Hyperlaxity or hypermobility is a common condition defined by an increased joint range of motion and distractibility a condition termed benign joint hypermobility syndrome (BJHS) [[1], [2], [3]]. The clinical diagnosis of BJHS is based on the Beighton score, but shoulder hypermobility is not always associated with this syndrome [4]. Patients with hypermobile shoulders tend to have an increased risk of sport-related injury and have an increased frequency of labroligamentous lesions associated with increased humeral head translation and recurrent subluxation/luxation [4]. There is also an increase in recurrence rates after glenohumeral instability repair in these patients [5]. Shoulder hypermobility has been linked to various conditions such as unidirectional or multidirectional instability, glenoid dysplasia, and rotator cuff impingement being an important factor in the surgical decision-making process [1,6,7]. Shoulder hypermobility may also influence imaging acquisition protocols (e.g., abduction and external rotation – ABER sequences, arthrography, etc.) and the interpretation of shoulder MRI and CT arthrography studies.

The clinical diagnosis of shoulder hypermobility is based on various well-documented clinical tests such as the sulcus sign, drawer test, shoulder external rotation over 85°, and hyperabduction tests [8,9]. However, when the clinical scenario is not suggestive or physicians are not sufficiently aware of the importance of this condition, shoulder hyperlaxity may be overlooked as there are no clear diagnostic criteria in conventional imaging studies. When arthrography is performed in the ABER position, imaging signs assessing capsular redundancy and laxity have been described and shown to be correlated with patients with multidirectional glenohumeral instability [10]. However, information on capsular morphology and other imaging findings in the presence of shoulder hypermobility is scarce. Quantitative imaging criteria could improve the identification and grading of glenohumeral hypermobility with potential surgical implications in addition to preventing interpretation and lesion identification errors when clinical hyperlaxity signs are unknown to the radiologist.

Glenohumeral hypermobility may influence the morphology of the glenoid as a reduced tension generated by capsular structures could lead to changes in the compression force of the humeral head to the glenoid [11,12]. This could explain differences in the morphology and version of the glenoid bone. We hypothesize there is a difference in the glenoid articular surface concavity in patients with and without shoulder hypermobility diagnosed clinically. This information could be useful for the diagnosis of hypermobility-associated injuries in patients without a prior diagnosis of shoulder hypermobility with potential clinical and acquisition protocol implications.

## 2 Materials and methods

### 2.1 Population

This study followed the STrengthening of the Reporting of OBservational studies in Epidemiology (STROBE) guidelines. From January 2017 to June 2021, shoulder CT and CT-arthrography study reports of 4269 patients acquired in our institution were reviewed retrospectively. These patients had been referred for the evaluation of shoulder pain. CT study reports were searched for the terms “hyperlaxity”, and “hypermobility” and derived words. Then based on this pre-search, the patient's medical records were reviewed for clinical signs of shoulder hyperlaxity. After this process, 50 patients with well-documented clinical signs of shoulder hyperlaxity evaluated by an orthopedic surgeon or by an examination performed by a radiologist from our center were selected and composed the hyperlax group. Glenohumeral hyperlaxity was defined by the presence of both of the following criteria: the presence of a sulcus sign (e.g., subacromial skin depression when downward traction is applied to the elbow of the patient with the arm relaxed to the side of the patient) and passive shoulder abduction range over 90° (e.g., evaluated with the elbow flexed and arm at the side of the patient) [13,14].

The medical records of patients with a normally appearing shoulder CT-arthrography were reviewed to confirm the absence of shoulder instability, hyperlaxity, and morphologic humeral changes. All of the included patients had a clear statements in their medical records stating a negative clinical exam regarding hypermobility and instability.

Following this procedure, a control group of 54 patients was formed. Thus, the final study population was composed of 104 patients. CT-arthrography was available for 86 patients and 18 had only conventional CT images available.

Patients with major glenohumeral degenerative joint disease, prior shoulder surgery, or fractures affecting the glenoid articular surface morphology were excluded. Patients with missing or incomplete medical records were not included.

In our institution, retrospective studies with anonymized data analysis do not require ethics committee approval (institutional review board waived).

### 2.2 Image acquisition technique

CT was performed on 320-detector row CT scanners (Aquilion ONE and Aquilion Precision Canon Medical Systems, Otawara, Japan). Intra-articular contrast injection (Iodixanol, Visipaque 270 mg, GE Healthcare) was performed by a radiologist under fluoroscopic guidance. A rotator interval anterior approach was used and 8–12 ml of undiluted contrast medium was injected. Images were acquired with either a helical or volume mode with the patient supine and the shoulder in neutral position using the following parameters: FOV 180 mm, 520 × 520–1024 × 1024 matrix, 120 or 135 kVp depending on the patient's body habitus, 200–250 mA (150–187,5mAs effective), slice thickness 0.25–0.5 mm and tube rotation time 0.75 s. Iterative reconstruction with a bone kernel was systematically used. CT was performed within 30 min after intra-articular contrast injection.

Anteroposterior shoulder radiographs were available for all patients.

### 2.3 Data analysis and post-processing

CT images from all patients were randomly displayed and independently analyzed by two radiologists with three and six years of clinical experience with musculoskeletal imaging blind to the presence of shoulder hyperlaxity on a picture archive and communication system workstation (PACS Carestream v11.4, Carestream Health, USA).

**Fig. 1:**
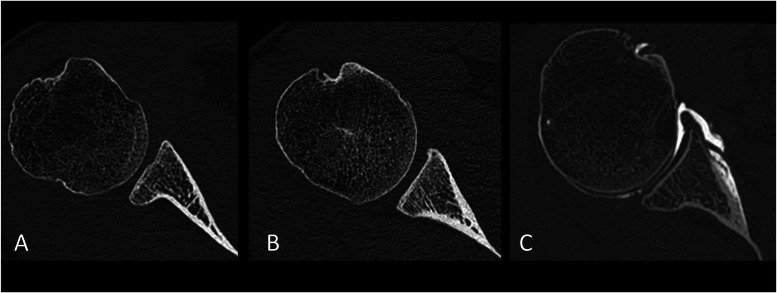
Subjective evaluation of the glenoid articular surface concavity on axial CT images at the mid-level of the glenoid. A) Convex glenoid articular surface in the right shoulder of a 21-year-old female with clinical hyperlaxity. B) Flat glenoid articular surface in the right shoulder of a 28-year-old male with clinical shoulder hyperlaxity. C) Concave glenoid articular surface in the right shoulder of a 54-year-old female with no signs of shoulder hyperlaxity.

Glenoid morphology was evaluated on an oblique axial image, orthogonal to the vertical axis of the glenoid, showing the largest diameter of the glenoid. Sagittal and coronal oblique reformats (thickness 0.5 mm) parallel to the glenoid plane were used to confirm the level of the glenoid's largest diameter. The morphology of the glenoid articular surface was subjectively evaluated and classified as concave, flat, or convex. In concave glenoid the center of the glene was deeper than the articular rims, it was at their level in flat glenoid articular surfaces and above in concave glenoid (Fig. 1).

**Fig. 2:**
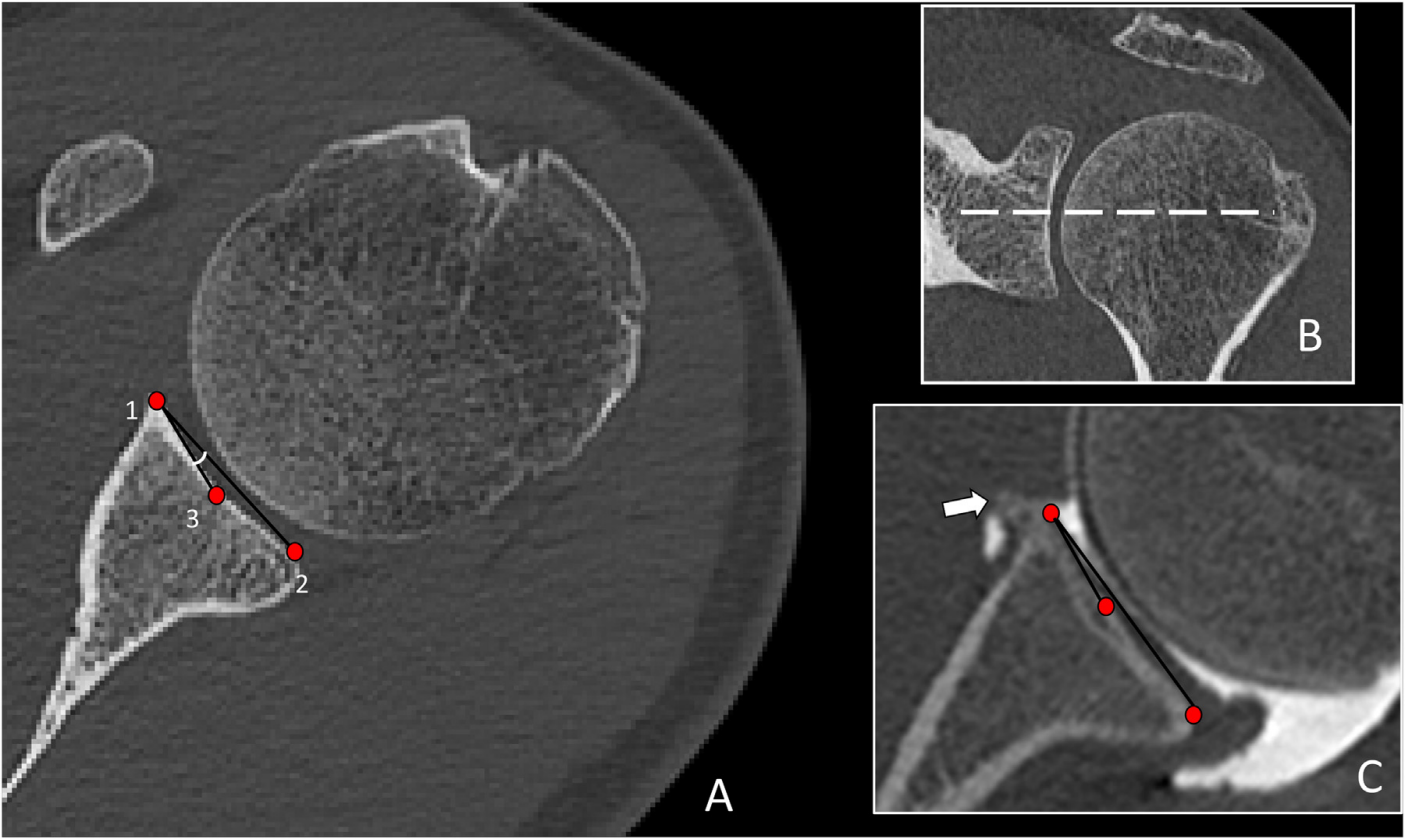
A) Glenoid concavity angle (GCA) measurement on an axial CT image passing through the center of the glenoid of a 33 year-old male with no clinical signs of shoulder hyperlaxity. The angle formed by the lines connecting points 1–2 and that connecting points 1–3 corresponds to the GCA. B) The dotted line on the upper left image shows the corresponding level in the coronal plane. In case of bone changes such as osteophytes, these are not taken in consideration when measuring the GCA. C) Correct measurement of the GCA on a 39 year-old patient with hyperlaxity, giving a GCA of 2° Osteophyte is visible at the anterior articular rim (arrow) and is not used for measurements.

The glenoid concavity, related to circumference and depth, was evaluated by measuring the angle between a line drawn between the anterior and posterior articular rims and another drawn between the anterior articular rim and the center of the glenoid, termed the glenoid concavity angle (GCA) [15] (Fig. 2). We utilized the largest diameter of the glenoid for this measurement, given that it typically corresponds to the area of maximum glenoid concavity. For the positioning of markers, we opted for the bony articular rims. This choice was motivated by their ease of identification in CT scans and their relatively lower morphologic variation compared to cartilage and labral tissue. However, in case of bone changes in the anterior or posterior articular rim such as osteophytes, these were not taken in consideration when placing the landmarks.

**Fig. 3:**
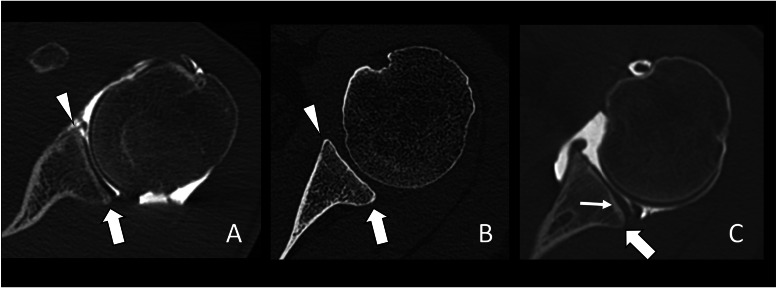
Assessment of the morphology of the posterior glenoid rim on axial CT images. A) 31-year-old male with a symmetric morphology between the anterior (arrowhead) and posterior (arrow) glenoid articular rims evocative of a normal glenoid morphology. B) 51-year-old male with a mild truncation of the posterior glenoid rim (arrow) compared to the anterior rim (arrowhead) evocative of mild glenoid dysplasia. C) 30-year-old female with moderate truncation of the posterior glenoid rim (arrow) with a posterior tilt of the glenoid surface (thin arrow) evocative of a mild glenoid dysplasia.

The morphology of the posterior glenoid rim was also evaluated at the level of the greatest diameter of the glenoid and was graded according to Harper et al.: no dysplasia (symmetric morphology between the anterior and posterior glenoid articular rims and labrum); mild (mild truncation of the posterior glenoid rim with a normal appearance of the labrum); moderate or severe (moderate or severe truncation of the posterior glenoid rim with a posterior tilt of the glenoid surface and a thickened posterior labrum) (Fig. 3) [16].

The critical shoulder angle (CSA) was also assessed as it is an indicator of the deltoid force vector on the glenoid articular surface [17]. The CSA is measured between the superior and inferior bone margin of the glenoid and the most inferolateral border of the acromion on anteroposterior shoulder radiographs.

### 2.4 Statistics

Statistical analysis was performed with the Jamovi software (version 2.3, 2022). Quantitative variables were presented as mean ± standard deviation (range). The Student's *t*-test was used to assess the variation of the GCA between patients with and without joint hyperlaxity. The ANOVA test was used to assess the association of the GCA with joint laxity and glenoid dysplasia. A *P* value of 0.05 was considered as the threshold of statistical significance. GCA interobserver variability was evaluated using the Shrout and Fleiss intraclass correlation coefficient (ICC) while kappa values were calculated to assess the interobserver variability for the diagnosis of glenoid dysplasia. Kappa and ICC values of 0 – 0.20 were considered to represent a slight agreement, 0.21 – 0.40 fair agreement, 0.41 – 0.60 moderate agreement, 0.61 – 0.80 substantial agreement, and 0.81 – 1 excellent agreement (33). Receiver operating characteristics curves (ROC) were generated, using the tools MedCalc® Statistical Software version 22.030 (MedCalc Software Ltd, Ostend, Belgium; https://www.medcalc.org; 2024), to evaluate the diagnostic performance of GCA values for the diagnosis of shoulder hyperlaxity. The cutoff values were selected using the smallest squared distance from the perfect classifier using the following equation: (1-Se)²+(1-Sp)².

## 3 Results

### 3.1 Population

The demographic characteristics of the study population in the hyperlax and control groups are presented in Table 1. The mean age was significantly higher in the control group (*P* *<* 0.0001). There was no significant difference in the sex ratio between the two groups (*P* = 0.70) There was no significant variation between CSA values between patients with and without shoulder hyperlaxity (*P* = 0.41). Mean CSA values were 32.8 ± 3.5 (19.9;37.5)° and 32.4 ± 3.2 (25.6;38.1)° in these groups, respectively. Glenoid dysplasia was identified in 32 (22 in the hyperlaxity group and 10 in the control group) by reader 1, and 32 (21 in the hyperlaxity group and 11 in the control group) by reader 2. The interobserver variability for the diagnosis of glenoid dysplasia was considered moderate (kappa = 0.41). Table 2 shows the distribution of all evaluated parameters in the study population.

**Table 1:** Population demographic characteristics in the subgroups studied.

	Controls	Hyperlax
H/F *(ratio)*	31/23 (1.3)	25/25 (1)
Age (years)	29.8 ± 9.4 (17;55)	38.9 ± 11.5 (18;62)
Laterality right *(n)*	25	18
Arthro CT *(n)*	40	46
CT *(n)*	14	4

*n*: number of patients; SD: standard deviation.

Age is presented as mean ± standard deviation (range).

**Table 2:** Distribution of all glenoid morphology parameters in the population subgroups.

		Hyperlax		Non hyperlax		*P* value		Kappa / ICC
		Reader1	Reader2	Reader1	Reader2	Reader1	Reader2	
GCA		2.3 ± 3.7 (−7;10)	2.3 ± 3.8 (−6 ;9)	6.6 ± 3.3 (−5;12)	5.3 ± 3.8 (5.6;13.5)	<0.0001	0.033	0.76
Glenoid morphology (*n*)	Concave	8	9	34	24	<0.0001	<0.0003	0.33
	Flat	31	21	19	25			
	Convex	11	20	1	5			
Dysplasia (*n*)		22	21	10	10	0.004	0.0016	0.35

*n*: number of patients; SD: standard deviation ; min: minimum; max: maximum.

GDA: Glenoid concavity angle (presented as mean ± standard deviation (range)).

### 3.2 Subjective analysis of the glenoid morphology

Glenoid morphology was significantly different between patients with and without shoulder hyperlaxity (*P* < 0.001 for both readers). The interobserver variability for the evaluation of glenoid morphology was considered fair (kappa = 0.3). For reader 1, in the hyperlaxity group, 42 patients (84 %) had a flat and convex glenoid, while 8 (16 %) had a concave glenoid. For reader 2 these figures were 41 (82 %) and 9 (18 %) respectively.

### 3.3 GCA assessment

The interobserver variability of GCA values was considered to be substantial (ICC = 0.65). There was a significant decrease in the GCA values in patients with shoulder hyperlaxity (2.26 ± 3.7 (−7;10)° and 2.3 ± 3.8(−7.6; 9)° for readers 1 and 2) indicating a shallower glenoid compared to controls (6.6 ± 3.3 (−5;13)° and 5.3 ± 3.8 (5.6; 13.5)° for readers 1 and 2)(*P* < 0.0001 for reader 1 and 0.033 for reader 2). The same was true when only patients without shoulder dysplasia were considered (*P* < 0.0001, for both readers). In patients with a non-dysplastic glenoid, the GCA was 3.6 ± 3.4 (−3;10)° in patients with shoulder hyperlaxity and 7.1 ± 2.5 (2;13)° in the control group for reader 1. For reader 2 these figures were 4.1 ± 7.9 (−2.3;9) and 5.9 ± 3.2 (1.2;13.5), respectively.

**Fig. 4:**
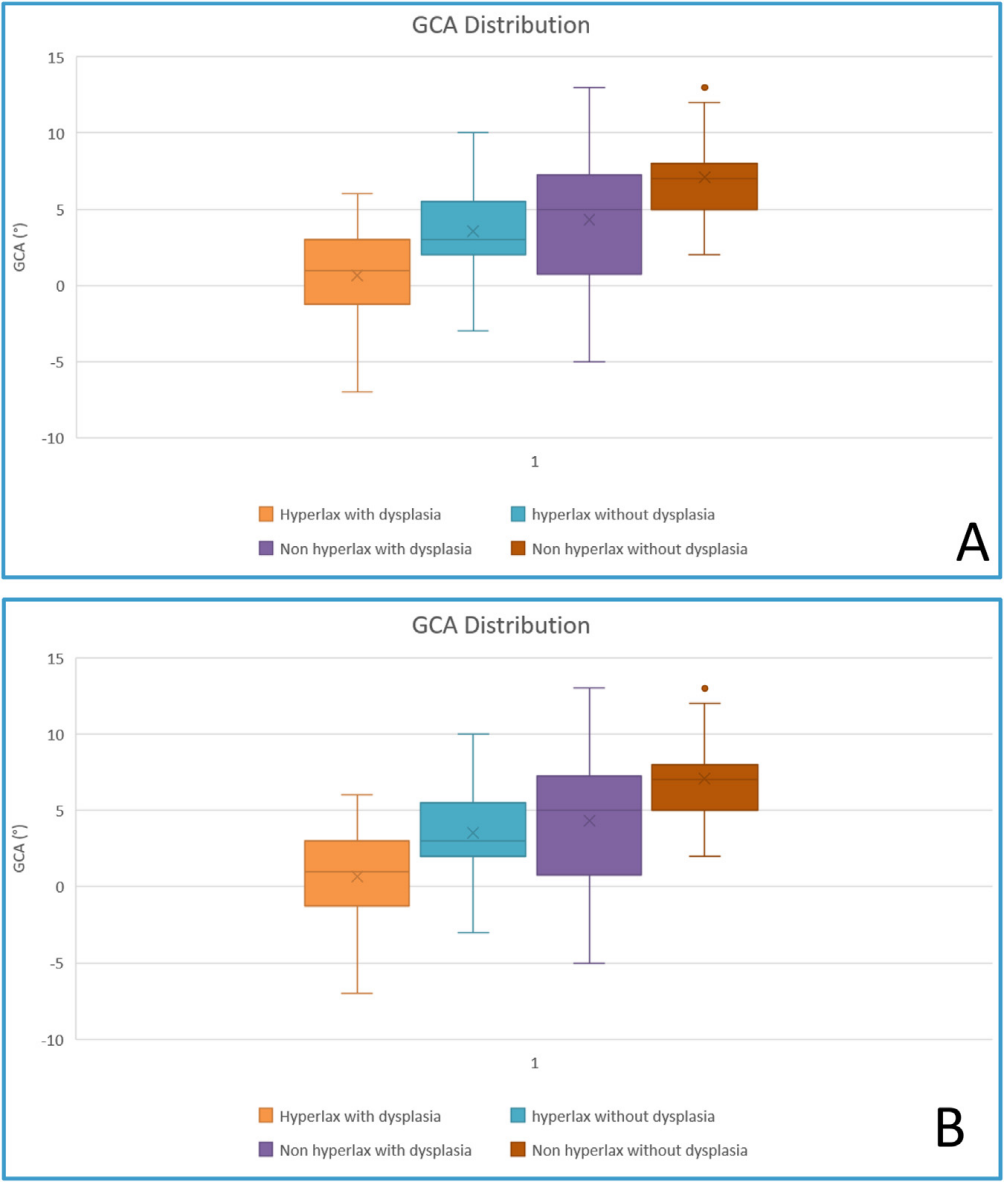
Box plot graphs demonstrating the variation of GCA values in all sub-groups studied for readers 1 and 2 (A and B respectively). Not the progressive decrease of GDA values from non hyperlax shoulders without dysplasia to hyperlax shoulders with dysplasia.

**Fig. 5:**
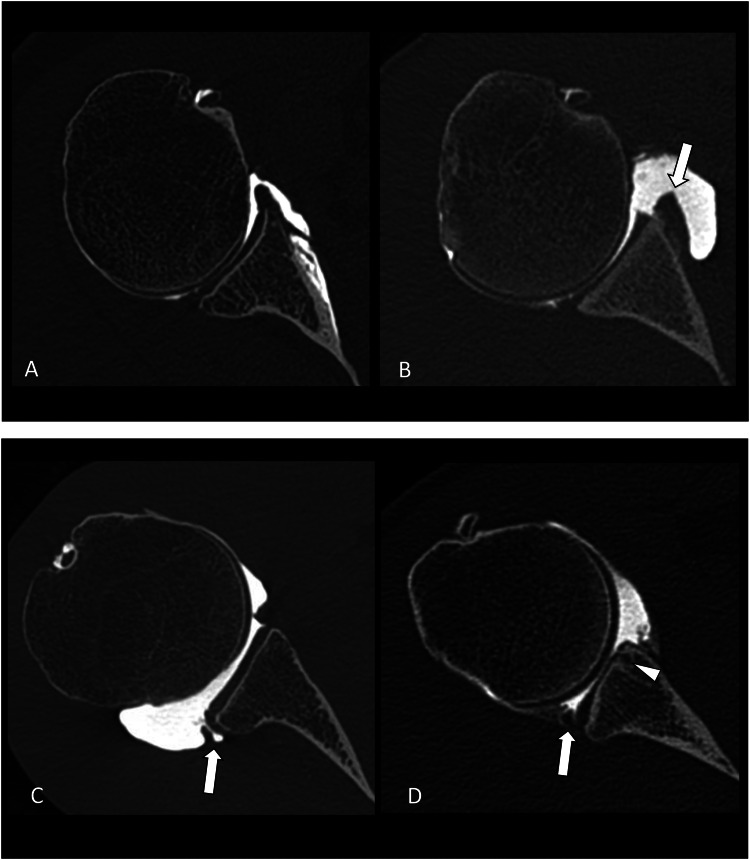
Variation of the glenoid concavity angle in the subgroups studied. A) Axial CT-arthrography image from the right shoulder of a 55-year-old female without shoulder hyperlaxity and no glenoid dysplasia. The glenoid articular surface was concave and the GCA was 11°. B) Axial CT-arthrography image from the left shoulder of a 23-year-old male with shoulder hyperlaxity and no glenoid dysplasia. The glenoid articular surface was flat and the GCA was 4° Note the signs of anterior shoulder dislocation and a chronic lesion of the anteroinferior labrocapsular structures (arrow). C) Axial CT-arthrography image from the right shoulder of a 36-year-old female with no shoulder hyperlaxity with mild glenoid dysplasia. The glenoid articular surface was flat and the GCA was −5° Note the associated intrasubstance tear of the posterior labrum (arrow). D) Axial CT-arthrography image from the left shoulder of a 30-year-old female with shoulder hyperlaxity and severe glenoid dysplasia. The glenoid articular surface was convex and the GCA was −7° Note the presence of glenoid chondral erosions and subchondral geode (arrowhead), as well as an intra-substance tear of a hypertrophic posterior labrum (arrow).

As expected the GCA was lower in patients with glenoid dysplasia compared to those without (*P* < 0.0001 for reader 1 and *P* = 0.0007 for reader 2). Mean GCA values were 1.78 ± 4.3 (−7;13)° in the hyperlax group versus 5.7 ± 3.3 (−3; 13)° in the control group for reader 1 and 0.59 ± 3.9 (−7.6; 11.9)° versus 5.2 ± 3,2 (−2.3; 13.5)° for reader 2. For both readers, there was a progressive decrease in GCA values from non-hyperlax non-dysplasic patients to hyperlax non-dysplasic, to dysplasic non-hyperlax to dysplasic and hyperlax (Fig. 4). For reader 1 these differences were significant for all comparisons (*P* < 0.001) except hyperlax non-dysplasic patients versus non-hyperlax dysplasic patients and non hyperlax dysplasic patients versus non-hyperlax non-dysplasic patients (*P* = 0.9 and 0.076, respectively). For reader 2, these differences were significant for all comparisons (*P* < 0.001) except non-hyperlax dysplasic patients versus hyperlax non-dysplastic, and non-hyperlax non-dysplasic patients versus hyperlax non-dysplasic patients (*P* = 0.5 and 0.072, respectively) (Fig. 5).

### 3.4 GCA performance assessment

GCA values presented a good diagnostic performance for the identification of shoulder hyperlaxity with ROC area-under-the-curve (AUC) varying (0.84 and 0.69 for readers 1 and 2). Using a cut-off of 4° the sensitivity and specificity values were 81 % and 82 % for reader 1 and 77 % and 55 % for reader 2. With a cut-off of 3°, the sensitivity and specificity values were 73% and 90% for reader 1 and 56 % and 62% for reader 2. With a cut-off of 5°, the sensitivity and specificity values were 83% and 67% for reader 1 and 81 % and 48% for reader 2

## 4 Discussion

There was a significant association between clinical signs of shoulder hyperlaxity and glenoid articular surface concavity estimated with the GCA on CT (*P* < 0.03). Shoulder hyperlaxity was associated with a shallower glenoid and 77–82 % of patients with GCA under 4° presented clinical shoulder hyperlaxity. Lower cut off didn't seem as relevant for screening and detection of hypermobility; the diagnosis remaining clinical. GCA interobserver reproducibility was considered substantial, measurements do not necessarily require arthrography to be performed as all landmarks used were bony landmarks easily identified on conventional CT. It is also likely that the same landmarks can also be identified in CT-like MR sequences [18]. Similarly, flat or convex glenoids were also significantly more frequent in patients with shoulder hyperlaxity but the reproducibility of subjective analysis was considered moderate and lower than of GCA. Glenoid morphology is highly variable among individuals and influenced by various factors (e.g., genetics, age, sex, peri‑articular soft tissue, etc.) [19]. Soft-tissue balance around the glenoid is particularly important and heavily influences glenoid morphology [[19], [20], [21], [22]]. Additionally, morphologic changes including glenoid concavity were associated with multidirectional and recurrent glenohumeral instability [23,24]. There is no consensus on the method of assessing the glenoid concavity and most studies used a subjective analysis method. Contrary to the presented results, prior studies reported excellent reproducibility for the subjective analysis of glenoid shape [23,24]. This difference could be related to a patient selection bias as these studies evaluated patients who underwent shoulder arthroscopy or had a prior history of shoulder dislocation, who are likely to have more prominent glenoid morphologic changes compared to patients with shoulder pain and joint hyperlaxity evaluated herein.

As shoulder hyperlaxity may have a variable and non-specific clinical presentation, CT studies showing a low GCA should warrant a search for clinical signs of shoulder hyperlaxity as this may have therapeutic implications as patients with shoulder hyperlaxity may require inferior capsular shift, do not respond well to nonoperative treatment and may be associated with high recurrence rates after Bankart repair [25]. Similarly, low GCAs should trigger an active search for lesions associated with hyperlaxity and multidirectional shoulder instability, such as internal joint impingement, labral tears articular sided rotator cuff tears, which may require specific imaging protocols (e.g., MR or CT arthrography and acquisitions in abduction external rotation) [26].

Humeral stability is strongly associated with glenoid concavity, which has contributions of the bony glenoid, articular cartilage, and labrum [27]. Glenoid dysplasia, despite its various degrees, is characterized by a bony deficiency of the posteroinferior glenoid rim combined with hypertrophy of adjacent cartilage and labrum [16]. Although glenoid dysplasia is associated with multidirectional and posterior glenohumeral instability, it is fundamentally distinct from glenoid concavity, which is also a crucial factor for glenohumeral stability [28]. Glenoid dysplasia does have a strong influence on glenoid concavity and GCA values were particularly low when shoulder hyperlaxity was associated with glenoid dysplasia. GCA values were higher in patients with hyperlaxity without dysplasia and highest in controls suggesting this angle allows a quantitative assessment of the severity of glenoid morphologic changes.

Various limitations of this work need to be acknowledged. Firstly, the sample size was relatively small. Although shoulder hyperlaxity is frequent, most patients are asymptomatic or paucisymptomatic and do not require imaging [1,29,30]. Secondly, we did not evaluate associated other morphologic glenoid parameters (e.g., version, tilt, etc.) and the influence of age and sex. Thirdly, we did not evaluate glenoid morphology and GCA on MR imaging. Further studies are required to ascertain if GCA measured on MRI, particularly on CT-like MR sequences has the same clinical implications [[31], [32], [33]]. Fourthly, the influence of articular cartilage and labrum on glenoid concavity was not assessed as cartilage thickness cannot be evaluated on conventional CT. Finally, other previously described imaging signs of shoulder hyperlaxity such as humeral head relation to the glenoid, morphology of the anterior band of the inferior glenohumeral ligament, rotator interval widening, etc. were not assessed [10].

## 5 Conclusion

GCA measurements were significantly reduced in patients with shoulder hyperlaxity compared to controls and in patients with glenoid dysplasia indicating an association between this angle and the severity of glenoid morphologic changes. The importance of GCA assessment is twofold as it can help identify unsuspected hypermobility and also provide a quantitative evaluation of glenoid concavity with substantial interobserver reproducibility. Thus, the identification of GCA values under 4° should warrant a search for clinical signs of shoulder hyperlaxity and associated rotator cuff and labral lesions, with potential implications in patient management.

## Disclosure

During the preparation of this work, the author(s) used Chat GPT 4 from OpenAI in order to improve readability and language. After using this tool/service, the author(s) reviewed and edited the content as needed and take(s) full responsibility for the content of the publication.

## Ethical statement

Hereby, I Sirine Hamitouche consciously assure that for the manuscript “Glenoid morphology variation between patients with hypermobile shoulder joints and controls: Identification of hyperlaxity-related morphologic bone changes” the following is fulfilled:

1) This material is the authors' own original work, which has not been previously published elsewhere.
2) The paper is not currently being considered for publication elsewhere.
3) The paper reflects the authors' own research and analysis in a truthful and complete manner.
4) The paper properly credits the meaningful contributions of co-authors and co-researchers.
5) The results are appropriately placed in the context of prior and existing research.
6) All sources used are properly disclosed (correct citation). Literally copying of text must be indicated as such by using quotation marks and giving proper reference.
7) All authors have been personally and actively involved in substantial work leading to the paper, and will take public responsibility for its content.

I agree with the above statements and declare that this submission follows the policies of Solid State Ionics as outlined in the Guide for Authors and in the Ethical Statement.

## CRediT authorship contribution statement

**Sirine Hamitouche:** Data curation, Formal analysis, Visualization, Writing – original draft, Writing – review & editing. **Fatma Boubaker:** Data curation, Formal analysis, Visualization, Writing – review & editing. **Gabriela Hossu:** Conceptualization, Formal analysis, Supervision, Validation, Writing – review & editing. **François Sirveaux:** Conceptualization, Supervision, Validation, Visualization. **Romain Gillet:** Supervision, Validation, Visualization, Writing – review & editing. **Alain Blum:** Conceptualization, Supervision, Validation, Visualization, Writing – review & editing. **Pedro Augusto Gondim Teixeira:** Formal analysis, Investigation, Methodology, Project administration, Supervision, Validation, Writing – original draft, Writing – review & editing.

## Declaration of competing interest

The authors declare that they have no known competing financial interests or personal relationships that could have appeared to influence the work reported in this paper.
